# Glanders: an overview of infection in humans

**DOI:** 10.1186/1750-1172-8-131

**Published:** 2013-09-03

**Authors:** Kristopher E Van Zandt, Marek T Greer, H Carl Gelhaus

**Affiliations:** 1 Battelle, 505 King Ave, Columbus, OH 43201-2693, USA; 2 MRIGlobal, 425 Volker Blvd, Kansas City, MO 64110, USA

**Keywords:** Burkholderia, Mallei, Aerosol, Biodefense, Animal models, Transmission, Infectious routes, Laboratory acquired infection

## Abstract

Glanders is a highly contagious and often fatal zoonotic disease, primarily of solipds. In the developed world, glanders has been eradicated. However, prior use of *B. mallei* as a biological weapon and its high mortality in inhalation animal studies has affirmed *B. mallei* as a biodefense concern. This threat requires the development of new glanders medical countermeasures (MCMs), as there is a lack of an effective vaccine and lengthy courses of multiple antibiotics needed to eradicate *B. mallei*. Here, we present a literature review of human glanders in which we discuss the clinical epidemiology and risk factors, potential routes of exposure, symptoms, the incubation period, and specific diagnostics. This review focuses on pulmonary glanders, as this is the most likely outcome of a biological weapons attack. Additionally, we outline current treatment regimens and propose a clinical definition of human pulmonary glanders infection.

## Introduction

Glanders is a highly contagious and often fatal zoonotic disease primarily of solipeds such as horses, mules, and donkeys. It was first described by the Greeks in 450–425 BC and again by the Romans in 400–500 AD. Throughout history glanders has been known by other names including equinia, malleus, droes, and farcy [1-5]. Glanders is primarily characterized by ulcerating lesions of the skin and mucous membranes. Solipeds are the natural reservoir of *Burkholderia mallei*. Donkeys are prone to develop acute forms of glanders while horses are more likely to develop chronic and latent diseases. Mules are susceptible to acute and chronic forms of the disease as well as latent infections [6-8]. *B. mallei*, the etiological agent of glanders, is a Gram-negative, non-motile, facultative intracellular pathogen. At one time, *B. mallei* infections occurred worldwide, but over the last 100 years the occurrence of glanders has decreased with the reduced economic reliance on solipeds as the primary mode of transportation, the implementation of testing all solipeds for glanders, and euthanizing those that are confirmed positive. The last naturally occurring human case in the United States was reported in 1934 [9]. Glanders in solipeds and humans has also been eradicated from Canada and Western Europe [10,11]. However, sporadic infections of animals are still reported in Far East Asia, South America, Eastern Europe, North Africa, and the Middle East [12-14]. Although human epidemics have not been recorded, isolated outbreaks in human populations and the deliberate use of *B. mallei* as a biological weapon have been documented [15,16].

Although glanders has been eradicated from many parts of the world, the threat of *B. mallei* being used as a weapon is very real. In fact, *B. mallei* was one of the first biological warfare agents used in the 20th century, specifically during World War I [1]. These malicious attacks on the opposing force’s animals were designed to impair the movement of troops and artillery, ultimately sabotaging a campaign. The former Soviet Union was also alleged to have used weaponized *B. mallei* against opposition forces in Afghanistan between 1982 and 1984 [2]. While the United States began work on biological warfare agents in the 1940’s including *B. mallei*, it did not weaponize *B. mallei*. However, during this work, seven individuals working in the US labs became infected. *B. mallei* has been considered a potential threat since the 1940’s because of its high infectivity, degree of incapacitation, and agent availability [5]. As further evidence that glanders is still considered a potential biological threat, the US Public Health Emergency Medical Countermeasures Enterprise convened subject matter experts at the 2010 HHS *Burkholderia* workshop in order to develop consensus recommendations for post-exposure prophylaxis against and treatment for *B. mallei*[17]. Glanders is relatively unknown in the West and its clinical symptoms in humans are nonspecific. Thus, diagnosis and treatment may be delayed. Therefore, we have reviewed the glanders literature to provide a description of human disease which will assist researchers developing medical countermeasures for glanders by creating a clinical definition against which animal models of human glanders can be qualified.

## Description of human glanders

### Clinical epidemiology and risk factors

Zoonotic transmission of *B. mallei* from solipeds to humans appears to be uncommon, even in cases of frequent and close contact with infected animals. However, rigorous examination regarding the rarity of transmission has not been conducted. Hypotheses to explain this low incidence include low organism concentrations at the infection sites or a high infectious dose required to cause disease in humans [9]. Despite the low incidence of animal to human transmission occupational exposure remains a key risk factor among veterinarians, veterinary students, farriers (hoof care workers), flayers (hide workers), transport workers, soldiers, slaughterhouse personnel, farmers, horse fanciers, and stable hands [9]. Subclinical infections in horses and mules also pose a hidden risk to humans, albeit a low risk. While infection by ingesting contaminated food and water has occurred, it does not appear to be a significant route of entry for human infections [18-20].

Human-to-human transmission is also rare. However, it may occur during occupational exposure in medical practice or at autopsies [3,9,19,21]. Transmission has also occurred in home settings, where the care of glanders-infected individuals has led to the infection of other family members [21].

While laboratory workers have rarely been infected, their close contact with high concentrations of virulent organism puts them at a higher risk for infection. Eight cases of laboratory acquired glanders have been reported between 1943 and the present at US research centers, specifically Fort Detrick, Maryland. Seven of the eight Fort Detrick laboratory-acquired infections occurred prior to modern biological containment practices [22]. The first six cases occurred during the performance of normal laboratory procedures that could generate aerosols such as washing, centrifugation, and aerating *B. mallei* cultures. In contrast to zoonotic transmission, aerosols of *B. mallei* are highly infectious [22]. Unfortunately, the seventh case is not well described in published literature. The eighth case may have resulted from inconsistent use of personal protective equipment particularly latex gloves [3,9,23].

### Routes of infection

Glanders is transmitted by direct invasion of abraded or lacerated skin; inhalation with deep lung deposition; and by bacterial invasion of the nasal, oral, and conjunctival mucous membranes. The occupational exposures described above most often occur through exposed skin, particularly the hands, arms, neck, and face [9]. *B. mallei* is not believed to penetrate normal intact skin, although wounds or penetrations during the likely exposure interval were not identified in many cases [9]. Indeed, most laboratory-acquired infections are not associated with injury or a recollection of injury [24]. In the case of the eighth case mentioned above, a break in the skin or a specific exposure-associated laboratory incident (needle stick or broken glassware) was not recalled or identified. However, this patient reported collecting a personal blood sample via a finger-stick for diabetic monitoring prior to entering the laboratory [9]. In light of the lack of glove use, the finger-stick site may have been the potential entry point of the bacteria. The clinical manifestation of unilateral axillary lymphadenopathy in this patient was consistent with percutaneous infection.

Six of the eight cases above were described in Howe and Miller’s report which detailed glanders cases occurring within one year among Fort Detrick laboratory personnel working with *B. mallei* (referred to as *Malleomyces mallei* in the report), including two notable cases [22]. Two workers (patients one and two) were present when a flask containing *B. mallei* was dropped and broken thus potentially creating aerosol droplets. Both patients were admitted to the hospital on the same day, approximately two weeks after the incident and were the only personnel present in the lab when the flask was dropped. The other four patients (patients three to six) were actively engaged in washing *B. mallei* growth from agar plates for the preparation of vaccines approximately 10–14 days prior to the onset of their symptoms. While all workers wore the required protective clothing at the time and used caution while performing their duties, the possibility that they inhaled an aerosol could not be eliminated. Patient four also failed to heat kill the *B. mallei* sample they were working with prior to making dilutions for turbidity measurements two weeks before symptom onset. Approximately two weeks before their admission to the hospital, patients five and six had engaged in procedures involving the aeration of cultures. They recalled that on at least two occasions, the containers had been opened immediately after the air current had been turned off, rather than after a period of delay long enough to avoid the escape of aerosolized organisms into the room. Additionally, these two patients were working with a strain of organism of higher virulence than the other four. These events strongly suggest aerosol route of infection by *B. mallei* can result in human glanders cases, especially in the laboratory setting [22].

### Time between exposure and symptoms

**Figure 1:**
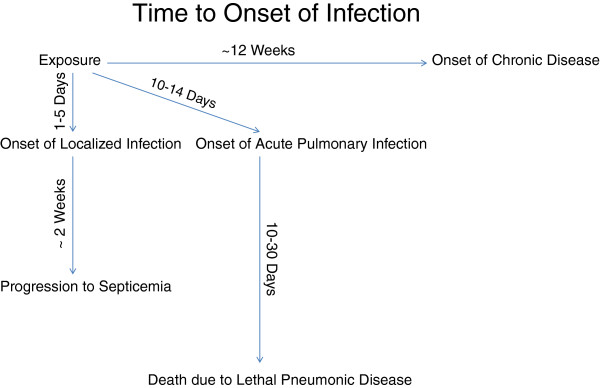
Time from exposure to symptoms.

The acute form of disease has a typical incubation period of 1–14 days [25], while the chronic form of the disease has an incubation period of up to 12 weeks. A localized infection typically follows within one to five days of exposure and may be characterized by swelling of the affected area and a weeping discharge. Acute pulmonary infections may require anywhere from 10–14 days of incubation before symptoms appear [25]. Septicemia may develop immediately after exposure or up to two weeks after initial infection. Pneumonic disease usually has a rapid onset and is almost uniformly lethal between 10 and 30 days in untreated cases [22]. Figure 1 summarizes the time between exposure and symptoms. An important feature of the eight cases that have occurred since 1943 is that at least half of the patients not only felt better but also experienced improved clinical signs for a period of time after the first wave of symptoms and prior to a second wave of symptoms. This period of temporary improvement has the potential to be misinterpreted as eradication of disease by patient and physician. Such temporary improvement should not limit recommended treatments.

### Diagnostics

The definitive diagnosis of glanders requires isolation of the organism and positive identification. Radiology may reveal abcesses in multiple organs including lungs, liver, and spleen, but these abcesses are indistinguishable from those caused by other disease and specific diagnosis is required [26]. While there is no validated *in vitro* diagnostic test for glanders, experimental serological tests, such as agglutination and complement fixation tests [27-29], and PCR based tests [30] have been used on a limited and experimental basis in solipeds as an alternative to isolating the organism. An indirect hemagglutination assay (IHA) used to diagnose melioidosis in endemic regions can also be used to diagnosis glanders [31]. Briefly, the IHA uses *Burkholderia* antigens to coat sheep red blood cells prior to their incubation with serum from suspected melioidosis (or glanders) patients. In the resulting reaction the antigen-coated sheep RBCs agglutinate or form a pellet if the patient is seropositive for *B. pseudomallei* or *B. mallei* (i.e., possesses antibodies to these pathogens) [31,32]. Since antibodies from glanders and melioidosis patients cross react with antigens present on a variety of *Burkholderia* species, the IHA assay is not a specific diagnostic test and other glanders-specific diagnostic tests are needed [31,33]. The mallein test is used for veterinary diagnostic purposes and involves injecting purified protein derivatives intradermally to observe a delayed type hypersensitivity reaction, similar to the tuberculin test for tuberculosis [34]. However, this test is not used in human diagnostics.

### Features of human clinical glanders

#### General symptoms

Many forms of glanders have been described, including chronic, disseminated, pulmonary, and septicemic. The variety of infections is largely explained by various routes of infection. Localized infections are generally regionally confined and typically characterized by foci of suppuration. The abscesses can ulcerate and drain for long periods of time. However, localized infections may disseminate, leading to pulmonary, septicemic, or multi-tissue infection [9]. The most common clinical features of the eight laboratory-acquired infections from Fort Detrick included (in order of most common occurrence) afternoon to evening low-grade fever, malaise, fatigue, headache, myalgias including backache, lymphadenopathy, and chest pain. Approximately half of the patients not only felt better but were also clinically better for a period of time after the first wave of disease symptoms. This period lasted from a few days to 2 months then patients developed clinical signs of infection.

#### Mucosal involvement

Involvement of the eye and conjunctiva in a *B. mallei* infection presents with excessive lacrimation and photophobia. Nasal involvement is characterized by inflammation and swelling of the nose, which is common following inhalation of *B. mallei*. This may be followed by copious nasal discharge. Additionally, infection may invade the nasal septum and bony tissues, causing fistulae and tissue destruction [9]. The face may swell and regional lymph nodes may become inflamed. Infection may also extend lower in the respiratory tract, resulting in bronchitis which can be accompanied by cough and mucopurulent sputum production. Constitutional signs and symptoms such as fever and chills typically occur within the first few days following infection. Additionally, these symptoms may persist through treatment and be severe. Common signs and symptoms can include, but are not limited to, fever or low-grade fever in the afternoon to evening; chills with or without rigors; and severe headache [9].

#### Cutaneous involvement

Cutaneous manifestations include papular lesions that may erupt anywhere on the body with a more chronic, indolent course of infection. *B. mallei* entry through an abrasion is typically followed by an inflammatory response, including pain and swelling. In these cases, a glanders node may appear as a single blister, gradually developing into an ulcer that may become hemorrhagic [18,35]. A localized infection with a discharge typically develops at the entry site. Inflammation may extend along regional lymphatics and cause lymphangitis with numerous foci of suppuration along their course. The endotoxins present in *B. mallei* strains affect the smooth muscle of the lymphatics [9] by enhancing the irritation and inflammation seen in the lymphatics.

#### Pulmonary involvement

A pulmonary infection typically results in pneumonia, pulmonary abscess, pleuritis, and plural effusion. Signs and symptoms of pulmonary infection can include cough, dyspnea, chest pain, and mucoplurent sputum. Nonspecific signs and symptoms such as fatigue, fever (often exceeding 102°F), chills, headache, myalgias, lymphangitis, sore throat, pleuritic chest pain, cough, tachypnea, dyspnea, discharge, and gastrointestinal signs often accompany respiratory infections [9]. Many symptoms may take up to 2 to 3 weeks to develop. Nonspecific signs, such as dizziness, rigors, myalgia, nausea, night sweats, severe headache, tachycardia, weight loss, and mucosal eruptions are also usually present and may indicate a disseminated infection [9].

#### Dissemination of infection

Dissemination from local cutaneous or mucosal infection result in septicemia and the colonization of internal organs such as the spleen, liver, and lungs with the development of abscesses [9]. Ultrasonography and computed tomography may reveal multiple, small discrete abscesses in both the liver and the kidney [23]. These infections are typically associated with septic shock and high mortality.

## Treatment of human glanders

Because of the rarity of human glanders cases, limited information exists regarding the use of antibiotics for the treatment of infected humans. However, *B. mallei* infections have responded to antibiotic therapy despite slow recovery times after delayed diagnosis or disseminated disease. *B. mallei* has been reported to be susceptible to many antibiotics *in vitro*[36-43]. Because *B. mallei* is a facultative intracellular pathogen, aminoglycosides and other antibiotics that are incapable of penetrating host cells are most likely not useful for the treatment of infection [40,41,43]. Most *B. mallei* strains exhibit resistance to several antibiotics (Table 1), due in part to the exclusion of antibiotics from host cells [3,9,15,18].

**Table 1:** **Antibiotics to which *B. mallei *is commonly resistant *in vitro***[40,44]

**Antibiotic**	**Minimum inhibitory concentration 50% growth**	**Minimum inhibitory concentration 90% growth**
	**(μg/mL)**	**(μg/mL)**
Amikacin	32	64
Amoxicillin	64	>64
Ampicillin	64	64
Cefazolin	>64	>64
Cefotetan	32	32
Cefoxitin	>128	>128
Cefsulodin	>128	>128
Ceftriaxone	32	32
Cefuroxime	64	64
Clindamycin	>128	>128
Fosfomycin	>128	>128
Ticarcillin	64	>128

Antibiotic efficacy has been tested against glanders in solipeds, hamsters, guinea pigs, and monkeys [38,42,43,45-47]. In hamsters, sodium sulfadiazine effectively treated acute glanders while penicillin and streptomycin were not effective [42]. Additionally, doxycycline and ciprofloxacin have been tested as post-exposure therapeutics in hamsters infected intraperitoneally with 2 × 10^7^ CFUs of *B. mallei*[43]. This study demonstrated that doxycycline therapy is superior to ciprofloxacin therapy when administered up to 48 hours after infection. It should be noted that some of these hamsters had a relapse of symptoms two to three weeks after discontinuation of ciprofloxacin treatment and four to five weeks after discontinuation of doxycycline treatment [43]. Additionally, doxycycline was able to provide 70% protection to hamsters following a low dose (5 × 10^2^ CFUs or 16 LD_50_s) aerosol challenge [9,45]. All tested antibiotics were ineffective against a high dose (5 × 10^3^ CFUs or 160 LD_50_s) of *B. mallei* delivered by aerosol [45].

The majority of human glanders cases occurred before antibiotic treatment was available, and over 90% of infected people succumbed to disease [48]. Of the human glanders cases recorded since the 1940s, several have been treated successfully with antibiotics [9,22,23,49,50]. Sulfadiazine was used to successfully treat six of the previously discussed laboratory-acquired infections at Fort Detrick, as well as two additional cases of non-laboratory acquired glanders described in 1951 [22,49]. The seventh case was successfully treated with a tetracycline derivative, aureomycin. In the most recent case of laboratory-acquired glanders, the patient initially received a 10-day course of a first-generation cephalosporin but symptoms persisted for a few weeks. The patient then received a 10-day course of clarithromycin, but symptoms returned 4 days after treatment was stopped. The patient was then successfully treated with imipenem and doxycycline intravenously for 1 month followed by oral azithromycin and doxycycline for 6 months [9,23].

Given the lack of historical data, current treatment regimens for human glanders are not well understood at this point in time, and no FDA approved treatment or treatment regimen exists. An exhaustive review of current and experimental treatments for both glanders and melioidosis has been published, but this review also describes the lack of clinical information for glanders treatment [51]. However, in order to manage laboratory exposures, treatment recommendations have been made [52]. Symptomatic case treatment of glanders requires a concurrent two pronged approach consisting of both an intensive intravenous (IV) therapy and oral eradication therapy. IV medication options are imipenem, meropenem or ceftazidime with or without trimethoprim-sulfamethoxazole (TMP-SMX). IV dosing schedule should last a minimum of 10 days and may be longer depending on the severity of illness. Oral therapy timing is also dependent on the severity of the disease and may run 12 weeks to as long as 12 months in duration. The treatment of choice for oral antibiotic therapy options are TMP-SMX as noted above with or without a secondary oral medication of doxycycline. Alternate therapies for TMP-SMX resistant *B. mallei* or patient intolerance consist of doxycycline, macrolides, chloramphenicol, quinolones, or amoxicillin-clavulanate as noted in the Occupational Health Manual from the United States Army Medical Research Institute of Infectious Diseases (USAMRIID) [52]. Since *B. mallei* and *B. pseudomallei* are similar, it is postulated that drug therapy experiences should be similar as well. Thus, doxycycline, amoxicillin-clavulante and quinolones monotherapies are at a greater risk of relapse than TMP-SMX and chloramphenicol.

Dosing regimen recommendations are shown in Table 2.

**Table 2:** Treatment recommendations

**Intensive IV therapy**	**Oral eradication therapy**
Imipenem 25 mg/kg up to 1 g every six hours	TMP-SMX 8/40 mg/kg up to 320 mg/1600 mg every 12 hours
Meropenem 25 mg/kg up to 1 g every eight hours	Doxycycline 2.5 mg/kg up to 100 mg every 12 hours
Ceftazidime 50 mg/kg up to 2 g every six hours	Amoxicillin-clavulanate 500 mg every eight hours or 875 mg every 12 hours for adults

### Disease outcomes in humans

The mortality rate for the pulmonary form of glanders has been reported to be 90-95% without treatment and up to 40% with treatment. The reported mortality rate for the septicemic form of glanders is greater than 95% without treatment and as high as 50% with treatment. The mortality rate for the untreated cutaneous form of glanders is 90-95% if it becomes systemic and as high as 50% with treatment. In the case of chronic glanders, the mortality rate may be as high as 50% despite treatment [53]. It should be noted that the previously reported mortality rates are likely to be extrapolations from the related disease melioidosis and that lack of recent human cases makes determining mortality difficult. A brief period of apparent recovery is a common clinical feature that can easily lead to delayed treatment and complications. However, despite the fact that three of the eight U.S. laboratory patients experienced a two month delay in effective treatment they were all successfully cured. These high success rates may reflect the quality of supportive care each patient received and the availability of appropriate antibiotics. The most recent patient had disseminated disease, and required ventilator assistance as well as a prolonged course of multiple antibiotics before improving. This patient was treated with imipenem and doxycycline, and there was rapid improvement. After two weeks, the imipenem was replaced by azithromycin, and the patient completed a six-month course of treatment with azithromycin and doxycycline [9,23]. In order to improve care of glanders patients, particularly in a mass-casualty situation, well-controlled animal studies conducted under the FDA animal rule would provide scientific evidence to efficiently direct medical treatment.

### Clinical definition of human pulmonary glanders

The following first two criteria and at least one of last two criteria are proposed for diagnostic confirmation of acute pulmonary glanders infection in humans.

1. Development of constitutional symptoms such as fever, rigors, myalgias, fatigue, headache, severe malaise, and pleuritic chest pain [22].

2. Chest X-Ray exhibiting new onset of infiltrates either segmental or lobar which may appear as nodular opacities. May also exhibit cavitary lesions or abscesses not only in the lungs but virtually any remote sites such as spleen, liver, subcutaneous tissue and muscle.

3. Positive isolation of *B. mallei* organism from sites such as cavitary lesions or abscesses not only in the lungs but virtually any remote site such as spleen, liver, subcutaneous tissue, and muscle as well as secretions, sputum, blood, and urine [52].

4. Positive *Burkholderia* species non-specific IHA titer and if positive a follow-up four-fold titer increase [52,54].

## Abbreviations

MCMs: Medical countermeasures; IHA: Indirect hemagglutination assay; IV: Intravenous; TMP-SMX: Trimethoprim-sulfamethoxazole.

## Competing interests

The authors declare that they have no competing interest.

## Authors’ contributions

KVZ conducted the literature review and helped draft the manuscript. MTG developed the clinical definition of glanders and helped draft the manuscript. HCG helped draft the manuscript. All authors read and approved the final manuscript.
